# The Interplay between the Host Receptor and Influenza Virus Hemagglutinin and Neuraminidase

**DOI:** 10.3390/ijms18071541

**Published:** 2017-07-17

**Authors:** Lauren Byrd-Leotis, Richard D. Cummings, David A. Steinhauer

**Affiliations:** 1 Department Microbiology and Immunology, Emory University School of Medicine, Atlanta, GA 30307, USA; labyrd@emory.edu; 2 Department of Surgery, Harvard Medical School Beth Israel Deaconess Medical Center, Boston, MA 02215, USA; rcummin1@bidmc.harvard.edu

**Keywords:** influenza A virus, hemagglutinin, neuraminidase, glycan binding, virus-host interactions

## Abstract

The hemagglutinin (HA) and neuraminidase (NA) glycoproteins of influenza A virus are responsible for the surface interactions of the virion with the host. Entry of the virus is mediated by functions of the HA: binding to cellular receptors and facilitating fusion of the virion membrane with the endosomal membrane. The HA structure contains receptor binding sites in the globular membrane distal head domains of the trimer, and the fusion machinery resides in the stem region. These sites have specific characteristics associated with subtype and host, and the differences often define species barriers. For example, avian viruses preferentially recognize α2,3-Sialic acid terminating glycans as receptors and mammalian viruses recognize α2,6-Sialic acid. The neuraminidase, or the receptor-destroying protein, cleaves the sialic acid from cellular membrane constituents and viral glycoproteins allowing for egress of nascent virions. A functional balance of activity has been demonstrated between the two glycoproteins, resulting in an optimum level of HA affinity and NA enzymatic cleavage to allow for productive infection. As more is understood about both HA and NA, the relevance for functional balance between HA and NA continues to expand, with potential implications for interspecies transmission, host adaptation, and pathogenicity.

## 1. Introduction

Influenza viruses are members of the *Orthomyxoviridae* family and all share the genomic make-up of single stranded, segmented, negative sense RNA. The RNA basis and segmented nature of the genome allow for remarkable evolutionary plasticity. The virus is able to incorporate changes based on both the error-prone replicative mutability and gene segment recombination (reassortment), such that virus populations can rapidly evolve in response to environmental conditions, transmission prerequisites, and host immune pressure. Influenza A viruses (IAV) have a particularly wide host range, branching out from the natural reservoir of waterfowl to domestic poultry and to mammalian species, including but not limited to swine [1], seals [2], horses [3], and humans.

Circulating seasonal human influenza viruses are estimated by the World Health Organization to infect 5–10% of the adult population annually with a morbidity rate of 3–5 million and a mortality rate of 250,000–500,000 deaths [4]. Seasonal influenza infects during predictable periods within the calendar year based in part on temperature and humidity [5,6], and the elderly, very young, and immunocompromised segments of the population are at particularly high risk. Influenza pandemics, however, caused by antigenically novel strains, arise at unpredictable intervals and can occur outside the seasonal norms, sometimes affecting alternative subgroups of the population such as young healthy adults. For both seasonal and pandemic influenza, the infection is established in the upper respiratory tract, but disease severity can vary significantly depending on a range of viral and host factors. Certain strains of influenza, such as those responsible for the devastating pandemics in 1918–1919, have been associated with particularly high morbidity and mortality, possibly due to lower respiratory tract involvement, secondary bacterial infections, and/or a high degree of immune pathology [7,8,9]. In avian species, a major determinant of high pathogenicity involves polybasic insertion sequences in the hemagglutinin (HA) of certain strains that allow for cleavage activation of infectivity by proteases expressed intracellularly in various tissues of the host, facilitating systemic infection [10,11,12]. To date, such strains have been restricted to subsets of H5 and H7 subtype avian viruses, and, although examples of limited avian to human transmission by direct contact have been reported, these have been self-limiting [13,14,15,16]. The phenotypic traits related to transmission, host range, and pathogenicity are multifactorial and polygenic in character. While the complex interplay between viruses and their hosts remain underexplored, the critical role of the HA as a determinant of transmission and host range is universally appreciated. A growing body of evidence highlights the significance for optimal functional balance between the HA and its companion viral envelope protein, the neuraminidase (NA) in the context of entry and egress, and it is likely that this balance impacts the processes of transmission, host range and pathogenicity as well.

Influenza A viruses are identified by the subtype of the HA and NA proteins, based on the antigenic reactivity to polyclonal sera and sequence data [17]. Within the aquatic bird reservoir, there are 16 known HA subtypes and 9 known NA subtypes, with an additional two HA subtypes and NA-like subtypes attributed to bats [18,19,20]. The HA subtypes are divided into five clades that can be segregated into two groups based on sequence comparisons and structural characteristics. Group 1 is composed of subtypes H1, H2, H5, H6, H8, H9, H11, H12, H13 and H16, and Group 2 consists of subtypes H3, H4, H14, H7, H15 and H10 [21]. Of the 16 HA subtypes and 9 NA subtypes found in avian reservoir species, only the H1, H2, and H3 HA subtypes and N1 and N2 NA subtypes have circulated extensively in humans over the past century. These were introduced into human populations as pandemic strains in 1918 (H1N1), 1957 (H2N2), 1968 (H3N2), and 2009 (H1N1), and each circulated and evolved, causing seasonal human Influenza for many years once they established a foothold. Influenza viruses of many different subtypes have been known to cause sporadic human infections or outbreaks without establishing human lineages. Of note are H5 and H7, which are associated with highly pathogenic infection in birds and have shown some isolated human transmission, [13,14,22] and H9, which, though not associated with high pathogenicity, does pose a pandemic threat due to the ability for human adaptation and the novelty of the subtype [23].

Why is it that such a small subset of all possible HA and NA subtypes have become human viruses? Are they unique in their structural and/or functional properties, or does it actually take a rare coincidence of events and conditions for novel strains to arise, adapt, and transmit efficiently in humans? Continued human population growth and the widespread use of agricultural practices that optimize contact between avian hosts, domestic birds, mammals, and humans, raise the prospect that novel human pandemics may actually arise with increasing frequency in years to come. To understand the complex nature of influenza ecology and cross-species transmission, it is critical to explore in-depth the nature of HA and NA glycan recognition and the identity and distribution of natural glycan receptors in the cells and tissues of their avian, mammalian, and human hosts.

## 2. Influenza Virus and Host Receptor Interactions

**Figure 1:**
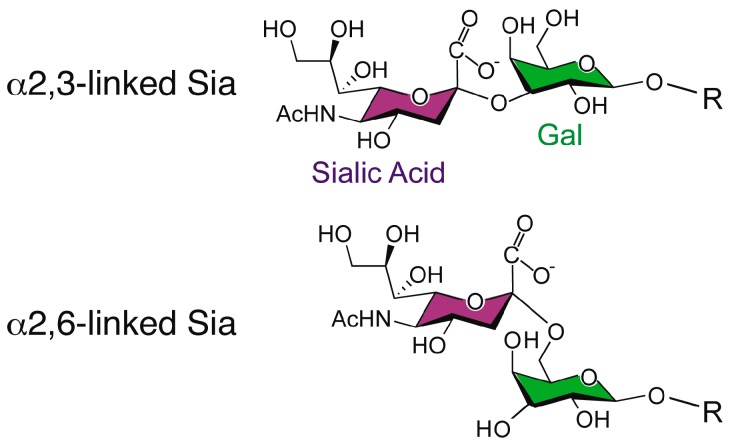
The structure of *N*-acetylneuraminic acid, a type of sialic acid, in both linkage conformations.

Influenza A viruses express HA on the surface of the virion in order to facilitate entry via receptor binding and fusion of the virion membrane with the endosomal membrane. Influenza viruses typically infect cells in the upper respiratory tract of humans, where the HA recognizes glycan structures terminating in N-acetylneuraminic acid (Neu5Ac), generally known as sialic acid, linked to galactose (Gal) in a β1-4 linkage to glucosamine (GlcNAc) (Figure 1) [24]. The linkage of the sialic acid to the penultimate galactose is considered to be a determinant of species specificity, with avian viruses characterized by binding α2,3-Sia and mammalian viruses by binding to α2,6-Sia [25,26,27,28,29,30,31,32,33,34,35,36,37,38,39,40,41]. This linkage preference, directed in part by structural features of the HA receptor binding pocket, is thought to correlate with receptor availability in the host [42,43,44].

**Figure 2:**
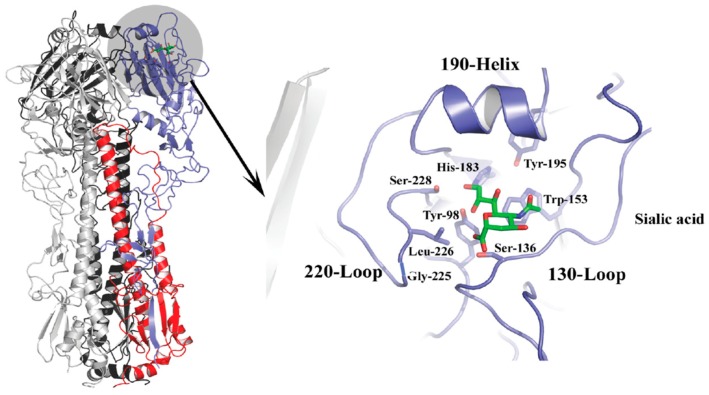
(Left panel) Trimeric HA, with one monomer colored gray, another black, and the third monomer shown in blue (HA1) and red (HA2). The location of the receptor binding pocket for the monomer shown in blue and red is highlighted by the gray circle, and this region is magnified and reoriented in the right panel to depict structural features and conserved residues in the binding pocket, along with the location occupied by bound sialic acid, shown in green. All numbering based on H3 subtype.

The HA spike protein is a membrane-anchored homotrimer composed of a fibrous stalk region, and a globular head containing a sialic acid binding pocket at the membrane-distal tip of each monomer (Figure 2). The binding site is characterized by four structural features, the 190-α helix, the 130-loop, the 220-loop, and a hydrogen-bonded network of conserved amino acids at positions 98, 153, 183, and 195 that constitute the base of the site [45,46]. The 130-loop contains residues 135, 136, and 137, which form main chain interactions with the sialic acid moiety of the receptor. The 220-loop contains residues for which mutations have been implicated in host specificity due to subtle changes in the architecture of the site that alter the interactions with different conformations associated with the glycosidic linkage type [39,47,48]. For example, human H2 and H3 strains containing leucine at position 226 display preferential binding to α2,6-linked Sia, whereas avian strains containing glutamine at this position favor α2,3-linked Sia. In these examples, the L226 widens the site changing the hydrogen bond network that would make contacts with the sialic acid whereas the Q226 does not [31,39,49]. The 190-helix also contains residues that have been implicated in determining specificity. Residues 190 and 193 have been shown to have importance for group 1 viruses, namely H1 and H5 strains [37,38,50,51], and changes at position 190 are sometimes accompanied by changes at position 225 as determinants of specificity. A number of other amino acid residues including but not limited to 189, 193, 194, 216, 198, 211, and 222 may influence the architecture of the region and have also been shown to be important for binding and specificity [38,52,53,54].

**Figure 3:**
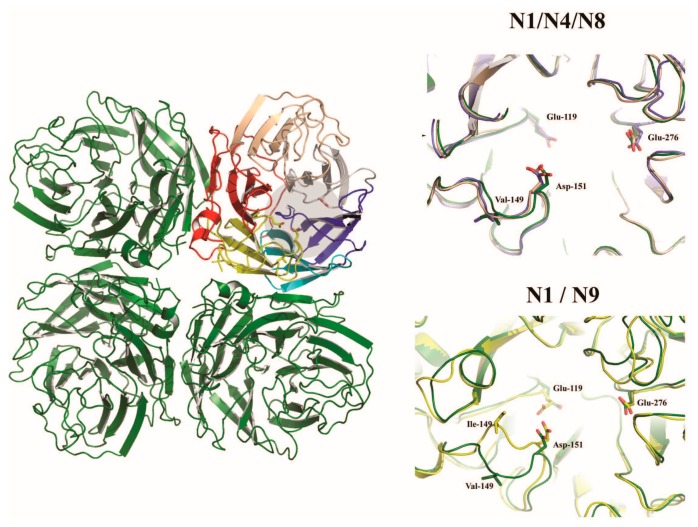
Influenza A virus neuraminidase homotetramer. The catalytic site is shaded in gray on the colored monomer in the left panel. The right panels illustrate subtype differences in the catalytic sites by superimposing the subtypes shown (N4 and N8—top panel; and N2 and N9—lower panel) onto the N1 subtype (shown in green).

The neuraminidase of influenza A virus is the receptor destroying enzyme in that it functions as a sialidase, cleaving sialic acid from cellular glycoproteins and also the viral glycoproteins that are being expressed in infected cells and assembled into virions. This action prevents HA mediated aggregation of nascent virus particles at the surface of the infected cell and allows for viral release [55]. The neuraminidase is a homotetrameric protein, with each subunit composed of a stalk domain supporting a head domain, comprised of six antiparallel β–sheets in a propeller-like arrangement (Figure 3) [56,57,58,59]. The active site is located within the head domain and is lined with conserved residues Arg118, Asp151, Arg152, Arg224, Glu276, Arg292, Arg371, and Tyr406 [56]. In addition to the enzymatic site, the head domain contains a calcium binding site [58,59] as well as a secondary Sia binding site found only in some strains [60,61]. The stalk domain is variable, though a shortening of the stalk often accompanies adaptation of IAV from waterfowl to poultry and has been linked to changes in the functionality of the NA and compensation in the avidity of HA [62,63,64,65,66,67]. Though mainly attributed to the release of budding virions, the NA has been speculated to have roles prior to cellular infection, trimming glycosylation of the HA [68] and cleaving potential inhibitory Sia from mucins [69].

Within the host, the environment is not necessarily favorable for viral dissemination and attachment as there are a number of innate host strategies in place to prevent infection. As an upper respiratory infection, the virus encounters the nasal passages first. Here, goblet cells express soluble mucins, and ciliated cells serve to move this mucus layer out and away from the surface of the cells [42,70]. The environment of the respiratory epithelia is complex. Surrounding and above the ciliated cells is a two-layer barrier. The periciliary liquid layer (PCL) is on the bottom and contains tethered mucin structures, while a more viscous gel-like mucus layer is on top. This arrangement has been described as a gel-on-brush model, and allows for the free beating of cilia underneath the mucus [71]. In addition to creating a scaffold in the PCL, mucins are also excreted and are the major components of airway mucus. Mucins, huge macromolecules, are composed of proline, serine, and threonine backbones with extensive glycosylation mostly with sialylated O-glycans [72]. MUC5ac and MUC5b are the two most abundant secreted mucins within the airway mucus and, while overexpression of MUC5ac appears to inhibit influenza virus infection, MUC5b appears to be essential for mucociliary clearance [73,74]. The tethered mucins, MUC1, MUC4, MUC16, and MUC20, not only function as a physical support, but also are important immune modulators, have roles in signal sequencing, and cell proliferation (reviewed in [75]). The virus must transverse the mucin layer and glycocalyx to reach an internalizable glycoprotein displaying the sialic acid receptor. Though much work has been done in relation to the morphology of the virus and the action of neuraminidase [76,77], little is known about virion transit to the cell’s surface prior to the receptor binding event. Nevertheless, the virus is able to initiate infection and appears to be relatively specific for certain cells types expressing certain sialic acid structures.

Influenza A virus particles are enveloped, and as such, lack a rigid outer capsid that creates a defining structure that would be consistent among the virions. The envelope is composed of lipid bilayer and is associated with four proteins, M1, M2, HA and NA. M1, the matrix protein, is thought to have the greatest effect on virion morphology, though it is not the sole determinant [76,78,79,80,81]. HA and NA transverse the bilayer and interact with the M1 protein. The matrix protein forms an inner core of the virion, giving structure and shape to an otherwise pleiomorphic lipid bilayer envelope [82]. The virus particles can adopt a range of morphologies ranging from rather spherical forms to extended filamentous structures, dictated at least in part by residues in the M1 protein [82,83,84,85,86,87,88]. Typically, spherical strains are found by electron microscopy in lab-adapted cultures, usually 80–100 nM in diameter, while most natural isolates are filamentous and vary in length [84,89,90]. It has been speculated that the filamentous shape allows for specific distribution of NA, with the protein clustered near the scission end of the filament to allow for NA cleavage of receptors at the infected cell surface [89,91]. This spatial arrangement could in theory also benefit transmission, as a cluster of NA at the leading end could be targeted to clearing mucins as the filamentous particle encounters the PCL. Electron micrographs have shown the spatial distribution in spherical particles seems to be more even for HA and NA around the exterior of the particle [91], perhaps indicating that in an environment intentionally favorable for replication, such as virus amplification in a laboratory, filamentous shape is not needed and may even be a hindrance. Changes in M1 that alter the virion morphology also seem to correlate with a change in the neuraminidase activity [76,87]. The interaction of NA and host mucins has been recognized since the 1940s, however the mechanism in the course of natural infection has yet to be fully elucidated [92,93]. Matrosovich et al., demonstrated that treatment with NA inhibitor, oseltamivir, diminishes influenza virus infection of differentiated and mucin producing human tracheobronchial cells and nasal epithelial cells indicating a role for NA prior to entry [69]. This result was recapitulated in undifferentiated cell cultures of A549 and MDCK cells [94]. Overlaying MDCK cells with human salivary mucins with mid to high Sia content reduces the rate of influenza infection. However, a porcine mucin overlay did not have the same effect with infection rates staying the same or increasing [95,96] even in the presence of oseltamivir or with a low activity NA. The inactivation of NA was expected to have an additive effect in combination with the mucus overlay, and so sustained infection may be due to differences beyond the sialidase activity and greater incorporation of Neu5Gc than Neu5Ac in human mucins. These combined results suggest that the mucin composition may contribute to the species barrier commonly attributed to availability of receptor type.

## 3. Carbohydrates Recognized by Influenza A Virus

**Figure 4:**
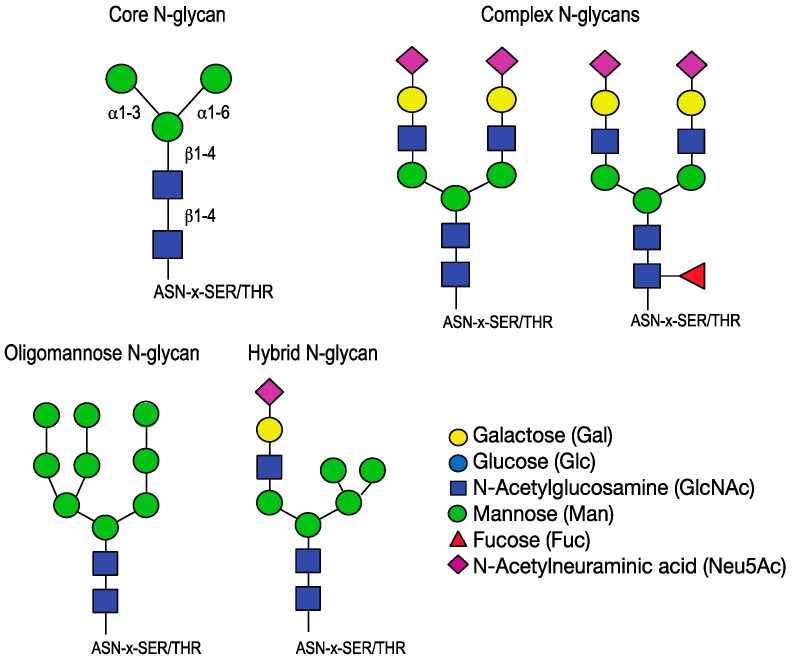
*N*-glycan core and complex structures. The core *N*-glycan is modified by the addition of subsequent monosaccharides to differentiate into oligomannose, complex or hybrid structures. Complex *N*-glycan structures relevant for IAV HA binding are shown.

Sialic acids (Sia), found at the terminal end of receptors utilized by influenza virus, are monomeric sugars with a nine-carbon backbone [93,97,98,99]. The term sialic acid broadly refers to this basic structure. This class of monosaccharides is one of the most diverse because of the potential for modifications. A small number of basic types of sialic acid exist, 2-keto-3-deoxy-d-glycero-d-galacto-nononic acid (KDN) and Neuraminic acid, with *N*-acteylneuraminic acid (Neu5Ac) and N-glycolylneuraminic acid (Neu5Gc) derivatives, though further modifications are found and expand the Sia repertoire to over 50 types [100]. Sialic acids are added to glycans in the Golgi by linkage specific sialyltransferases and are often found at the terminus of *N*-glycans, *O*-glycans, glycoproteins and glycosphingolipids. Sialic acids are important for many biological processes including cellular differentiation, growth, signaling and fertilization [100]. They are known to stabilize protein structures and can inhibit recognition or enhance it [101]. The terminal decoration of glycoproteins makes sialic acid an ideal target receptor for a variety of pathogens, including influenza viruses. When comparing carbohydrates isolated from the swine lung, *N*-glycans terminating in sialic acid are the preferred receptor for influenza viruses of human, avian and swine host strains [102]. *N*-glycans, distinguished by the covalent *N*-glycosidic bond to Asn-X-Ser/Thr, have a core structure of Manα1-6(Manα1-3)Manβ1-4GlcNAcβ1-4GlcNAc-Asn that can be further modified to generate a wide variety of structures. In general, three types exist: oligomannose, complex, and hybrid. Type 1, or oligomannose, structures contain branches comprised solely of mannose. Type 2, or complex-*N*-glycans, structures have branches attached to the mannose core. Type 3, or hybrid, structures have mannoses on the Manα1-6 of the core and a branch off of the Manα1-3 (Figure 4).

The synthesis of the *N*-glycans is mediated by membrane bound glycosidases and glycosyltransferases that are regulated by cellular conditions, such that two different cells may ultimately present a different range of glycans based on the cellular environment. The implications of this fluid state of glycosylation are significant for viral infection, as it is possible that some cells are more resistant to entry than others due to a myriad of host factors impacting the glycosylation pathways. The processing of the *N*-glycan occurs in the trans-Golgi and involves modifications to the core, elongation of the branches and the addition of terminating sugars such as sialic acid [103]. *N*-glycans serve many functions both intracellularly and as features of signaling molecules on the cell surface. It is known that *N*-glycans ensure proper folding of glycoproteins prior to exit from ER [104]. A number of congenital disorders of glycosylation, related to defects in *N*-glycan synthesis and presentation, have been identified and have developmental, neurological, gastrointestinal and immune effects indicating the importance of N-glycosylation [105]. Sialic acid is also found linked to *O*-glycans, a class of carbohydrates designated by the *O*-glycosidic bond of an *N*-acetylgalactosamine to a serine or threonine. *O*-glycans can have one of eight core structures, though core 1–core 4 are the most common, and also can undergo additional elongation and modification processes to generate a wide range of structures. As with *N*-glycans, *O*-glycans have many biological roles, from immune recognition to the hydration of mucosal layers in the epithelia [72]. These glycans, though not directly bound by influenza viruses, are a fundamental component in mucins and therefore may be significant for the virus interaction with the host environment.

## 4. Presentation of Receptors in Avian, Swine, and Human Hosts

The host range for influenza A virus is diverse, ranging from avian to a number of mammalian species. However, aquatic birds of the orders *Anseriformes* and *Charadriiformes* make up the natural reservoir. The *Anseriformes* include ducks, geese and swans while the *Charadriiformes* include shore birds, gulls and terns. Infection in these animals is gastrointestinal via an oral-fecal route [43,44,106] and is mostly asymptomatic, indicating an adaptation to the species. Though the virus recognizes the same general structure of receptor, a sialic acid terminating sugar, avian and mammalian viruses have been shown to recognize different sialic acid linkages. It is known that birds, pigs, and humans express sialic acid on cells at the site of influenza virus infection and much work has been done to identify the linkage type present. Most often lectin histochemical studies are done with the plant lectins *Sambucus nigra* and *Maackia amurensis*. *Sambucus nigra*, SNA, derived from elderberry bark, recognizes sugar structures terminating in α2,6-sialic acid (Neu5Acα2-6Galβ1-4GlcNAc). *Maackia amurensis*, MAA, from the seeds of the *Maackia amurensis* tree, contains two isoforms designated MAL (MAA-II) or MAH (MAA-I) that differ in glycan recognition. MAL binds α2,3-sialic acid (Neu5Acα2-3Galβ1-4GlcNAc) and some sulfated structures, while MAH recognizes a different motif (Neu5Acα2-3Galβ1-3) [107,108,109,110].

### 4.1. Birds

Multiple groups have studied the presentation of sialic acid within the avian respiratory and intestinal tracts with differing results. A comprehensive examination of the literature reveals that instead of firmly understanding the dominating sialic acid linkage present at the sites of infection, we are recognizing the variability, and therefore, limits of lectin histochemical studies. For example, an extensive study completed by França et al. of representative species of the two aquatic avian orders indicates that α2,3-sialic acid and α2,6-sialic acid is present throughout both the intestinal and respiratory tracts in almost all of the birds [111]. In a direct comparison of terrestrial and aquatic birds, Kuchipudi et al. determined that both chickens and ducks express α2,3-Sia in intestinal epithelial cells and that neither expresses α2,6-Sia [112]. This finding is contradicted by the aforementioned data on *Anseriformes*, the order encompassing duck species, but corroborates the results of others [113,114]. A brief overview of the current data is summarized in Table 1. Strict conclusions about the predominant Sia linkages become more obscure as tissue type and cell type are explored, with groups examining different tissues within the respiratory tract and differentiating between cell types, such as ciliated and non-ciliated epithelial cells. The two isoforms of MAA are problematic due to the differences in specificity and the fact that they are frequently distributed together or one isoform is not distinguished from the other commercially [115]. Even in more recent studies conducted after the resolution of the “MAA problem” and using both isoforms or just MAL, there are still discrepancies in results for avian hosts, potentially linked to the method of tissue presentation. In general, though, it appears that both linkage types are present in the tissues harboring virus infection.

### 4.2. Pigs

Swine have often been implicated in the zoonotic spread of influenza viruses. As discussed, the pandemic event of 2009 occurred when a reassortant swine virus crossed the species barrier to sustain infection in humans. Just as with avian strains, lectin studies have been used to examine the receptor distribution and similar discordant results have been reported (Table 1). The dogma for some time was that because pigs harbor both receptor types, the animal can serve as a mixing vessel allowing for an avian virus to infect via a 2,3 linked receptor and develop the ability to recognize a 2,6 receptor then transmit to humans [44,112,118,124,125]. Using lung explants, Punyadarsaniya et al. demonstrated both avian and porcine influenza virus infection in swine lung cells. They determined that ciliated and non-ciliated mucus producing cells exhibited α2,6-Sia and were readily infected by the swine virus (H3 subtype). Only ciliated cells stained with MAL, but both cell types were infected with an avian strain (H7 subtype) [126]. Van Poucke et al. report minimal MAL staining of the porcine trachea indicating a lack of α2,3-Sia receptors [127] or perhaps a limit of lectin recognition. Swine viruses typically display a greater 2,6 receptor binding specificity than 2,3 [25,26,33,102,120,121,128] which may indicate that though α2,3-Sia may be present in the porcine respiratory tract, it is not displayed on cells utilized in infection. Interestingly, in swine particularly, influenza viruses recognize the other species of sialic acid, *N*-glycolyneuraminic acid (Neu5Gc). Neu5Gc is prevalent in swine, but not common in humans [24,129,130,131,132]. Swine viruses that transmit and adapt to humans lose Neu5Gc recognition [25,133], and though studies show a preference of Neu5Gc binding for swine isolates [133], it is unclear if infection within the swine host is mediated by both sialic acid species or if a Neu5Ac specific virus, such as a human strain introduced during reverse zoonosis, would require further adaptation to efficiently spread in the porcine respiratory tract. The comparison of recent H3N2 swine isolates to reference human strains reveals distinct similarities between the two, leading to the characterization of the HA gene of the swine isolate as “wholly human” [134] and therefore indicating that modification of the receptor binding pocket to prefer Neu5Gc binding is either unlikely or not associated with residues known to alter linkage specificity.

### 4.3. Humans

As with swine viruses, viruses isolated from human infection display a α2,6-Sia receptor preference in receptor binding studies. An attempt to determine the localization of receptors with human respiratory tissue has fallen to the same disparity as avian and swine studies (Table 1). For example, it has been proposed by lectin histochemistry that the upper respiratory tract is populated with primarily α2,6-Sia linkages and the number of α2,3-Sia linkages increase as the lower respiratory tract is reached [42,115]. This finding is in contrast to the studies of Matrosovich et al., who determined that both receptor linkages were present on cultured airway epithelial cells and were differentiated by cell type, with ciliated cells expressing α2,3-Sia while non-ciliated cells expressed the α2,6-Sia presumably used for infection [135]. Some linkage identification has been done independent of lectin analysis and these methods involve glycomic analysis and PVA or pattern of virus attachment experiments. It was revealed by mass spectrometry analysis of glycan samples treated with linkage specific sialidases that both 2,3 and 2,6 linkage types are found in bronchial and lung explants [136]. PVA techniques use viruses as the histochemical agent instead of lectins and provide a direct stain of virus binding [122,123,137]. It was shown that human viruses, an H1N1 and an H3N2, bind more to ciliated cells than non-ciliated and are more localized to the trachea and bronchi than the bronchioles while avian viruses of subtypes H5 and H6 bound more in the alveolar region with less attachment to the trachea [123,137]. These experiments are informative, but only tangentially address the classification of the Sia-linkage. Viewed in conjunction with the afore-mentioned limitations associated with lectin histochemistry, it is clear that continued development of alternative approaches will be needed to fully illuminate the mechanisms behind the initiation of infection.

The technology used to directly examine the receptor binding properties of HA has expanded drastically from the original experiments based on the agglutination of erythrocytes [97,138]. Experiments with erythrocytes from animals with differences in Sia expression or with disialylated and then selectively re-sialylated RBCs reveal the linkage specificity between avian and mammalian HAs [139,140,141,142,143]. Beyond that, cell lines deficient in *N*-glycosylation pathways have been utilized to establish the necessity of sialylated *N*-glycans for entry [144]. Various methods of synthetic sialic acid presentation have been used to study HA, from the generation of synthetic Sia analogs to be used in ELISA formats or for bio-layer interferometry to the fixed presentation of synthesized glycans on a microarray [145,146,147,148]. The synthetic glycan microarray slides printed by the Consortium of Functional Glycomics (CFG) and others allow for the rapid characterization of receptor specificity for influenza isolates [25,26,38,147,149,150,151,152]. Though the CFG slides are extremely useful for determining broad specificity and for illustrating the structures that can be bound by influenza viruses, they are limited in the realm of biological significance. To address this gap, a novel technology of natural shotgun glycan microarrays was developed [153] and expanded to use the tissue of a natural host of influenza, swine, to supply the glycans [102]. The results of the swine lung shotgun glycomics study reveal that though avian and mammalian viruses generally display the broad receptor specificity illustrated by the HA assays and synthetic arrays, receptor preference was often much more selective, with only a subset of available sialylated glycans of either 2,3 or 2,6 linkage being bound by the viruses tested, suggesting that structural determinants beyond the terminal sialic acid are important for HA binding. Additionally, the pig lung data reveal that multi-antennary glycans are often recognized by certain strains that fail to bind to monoantennary glycans with the equivalent sugar chains [102], suggesting a mechanism for amplifying the multivalent virus-receptor interactions and increasing avidity. This phenomenom has recently been examined systematically using extended synthetic glycans comprised of poly-*N*-acetyl-lactosamine units, and a model for branched glycan interaction with two subunits of the same HA trimer, presumably resulting in increased receptor avidity, has been proposed [154].

The widely accepted but somewhat simplistic explanation for receptor binding specificity has been that the differences in sialic acid linkage type present at the site of infection correlate with the receptor specificity of avian and mammalian viruses. The interpretation has been that avian viruses are specific for α2,3- linked sialic acid because those structures predominate among glycans at the site of infection in the intestinal tract of birds and that human and swine viruses prefer α2,6-Sia because that is the predominant receptor in the respiratory tract. Based on the differential reports of various lectin studies, it is clear that a more heterogeneous mix of receptors is prevalent in all hosts than was originally thought. In addition, many groups suggest that certain species of birds and swine could be a mixing vessel for avian and human viruses to intermingle, swapping gene segments and potentially creating a human-adapted virus from the avian host [111,124,155,156]. If, as predicted, there is a prevalence of both receptor types at the site of infection, then what beyond availability leads to the receptor specificity attributed to the different species? To answer this question, a focused investigation into the cellular presentation of the glycans, including what glycoproteins they modify, and the localization and cellular function of those glycoproteins must be examined.

## 5. Interspecies Transmission

The genetic plasticity of influenza viruses allows for a wide host range and distinctive propensity for these viruses to cross species barriers, and interspecies transmission is not uncommon with multiple documented instances. Avian to avian transmission between wild waterfowl and domestic poultry has been widely reported and can often lead to huge economic losses either through infection by highly pathogenic strains or culling of the population to control the threat. Transmission from domestic poultry directly to humans has been shown to occur and has been linked with the use of live poultry markets in southeast Asia [157]. Swine hosts frequently play a role in interspecies transmission both as the recipient host of transmitted avian and human viruses and the donor for a human transmission event (reviewed in [116]). Swine are also implicated in reverse zoonosis, as human viruses jump to swine and circulate within the swine host before potentially returning to humans [117,134]. To date, most events have been relatively mild, with the exception of the pandemics, as interspecies transmission and sustained human circulation has been limited to viruses of low pathogenicity. A highly pathogenic virus has not been able to breach the species divide and transmit effectively between humans yet, however, it is likely that eventually this may happen and the effects could be devastating.

Although receptor preference might be the most significant and certainly the best known determinant of species specificity, the adaptations required for interspecies transmission are multi-factorial, and much is yet to be understood. Starting with the receptor itself, crystal structures have revealed that the position of the sialic acid, both linked 2,3 and 2,6, within the receptor binding site of an avian or human HA, respectively, is virtually superimposable [119]. The major differences observed between avian and human viruses and their cognate receptors involve the location and interactions of the glycan adjacent to the terminal sialic acid. In 2,3-Sia receptors, the glycans adopt an extended conformation and exit the pocket over the 220-loop, whereas in 2,6-Sia receptors the glycans fold back placing the third sugar above the plane of sialic acid [28,31,46,49,119,158,159,160,161,162]. The structures of the pentasaccharide LSTc in complexes with H1, H3, or H9 HAs reveal different conformations for the same 2,6-Sia receptor, depending on the HA. Though LSTc retained a folded structure when bound to each of the three HA subtypes, the residues contacted by the internal sugars varied from one complex to the other, and included residues in the 190-helix as well as the right side of the pocket [35]. These observations highlight the flexible nature of glycan receptors and illustrate that determinants beyond the terminal sialic acid contribute to binding. Indeed, determinants such as core fucosylation have also been shown to influence binding [102]. The features of the receptor binding pocket that mediate this receptor preference have been identified as residues 226 and 228 for H3 and H2 viruses and residues 190 and 225 for H1 viruses. A mutation at position 226 from a glutamine to a leucine is associated with a α2,3-Sia to α2,6-Sia binding switch, while at position 228, a glycine to serine switch is necessary [39,47,48,49]. For H1 subtypes, a glutamic acid to aspartic acid at residue 190 and an aspartic acid to glycine at residue 225 correlate with the receptor specificity switch [28,150,163,164]. Recent avian isolates have been observed with mammalian receptor binding traits. An H5N1 displaying an increase in α2,6-Sia specificity has been isolated in Egypt and may link to the rise of local direct-contact infections of humans with avian H5 [165,166]. In China, an H7N9 strain found with the mutation Q226L displays specificity for both α2,3-Sia and α2,6-Sia [22,161,167,168,169,170,171,172]. Although these viruses developed recognition of the mammalian receptor type, avian to human transmission was limited to cases of direct contact. A potential explanation entails a significant reduction in α2,3-Sia specificity in addition to acquisition of α2,6-Sia specificity for efficient transmission in humans, perhaps due to inhibitory properties of mucins in the human respiratory tract, which are rich in glycans containing α2,3-Sia [122,158,173]. In an experimental setting, both Herfst et al. and Imai et al. described a minimum of four mutations in HA, altering receptor specificity and removing a glycosylation site, that contribute to avian–mammalian respiratory droplet transmission, however sustained transmission was not attributed to changes in HA binding alone [174,175]. For successful interspecies transmission and efficient replication of the virus within the new host, other viral factors in addition to receptor switch must undergo adaptation to allow for the disparities in temperature and pH that accompany the new host environment [174,176,177,178,179,180,181,182,183].

## 6. Functional Balance of HA and NA

The two surface glycoproteins of influenza A virus, hemagglutinin and neuraminidase, mediate a range of host interactions from receptor binding to viral release. As mentioned previously, the hemagglutinin binds to carbohydrates on the cell surface terminating in sialic acid. Neuraminidase, likewise, acts on sialic acid, cleaving the moiety from both HA and cell surface glycans. Because they share a substrate, it seems obvious then that a functional balance between the two proteins would exist, preventing the work of one from overwhelming the role of the other. This functional balance has indeed been established in terms of binding avidity and neuraminidase enzymatic activity. Baum and Paulson recognized a drift in NA specificity to obtain some sialidase activity against α2,6-Sia from 1957 to 1987. Strains circulating prior to 1967 and 1968 had strict α2,3-Sia specificity, but recognition of α2,6-Sia gradually increased until finally, viruses isolated in 1972 exhibited equal sialidase activity against both α2,3-Sia and α2,6-Sia [184,185]. This time period saw the introduction of the H3 subtype following the 1968 pandemic and might indicate the selective advantage of establishing sialidase activity against potential receptor structures that could lead to a null infection. Data collected identifying the affinities of H3 HAs for α2,3-sialyllactose and α2,6-sialyllactose highlights a general increase in affinity of HA for α2,6-Sia from the time that the strain was introduced in 1968 [164]. Xu et al. compared the HA affinity to 6′-SLNLN, NeuAcα2–6Galβ1–4GlcNAcβ1–3Galβ1–4GlcNAc, to the NA activity against 4-MU-NANA, 2′-(4-Methylumbelliferyl)-α-d-*N*-acetylneuraminic acid, for pandemic strains from 1957, 1968 and 2009 and found a correlating increase in both [186]. This increase in affinity, if reflected biologically, could explain the rise in α2,6 sialidase activity of human strain NAs and highlights the intrinsic need for functional balance of the two proteins. This concept has been examined in a variety of laboratory studies with a forced imbalance of the two proteins and subsequent adaptation. Studies with oseltamivir led to escape mutants that exhibit resistance, not always by altering the NA, but in some cases by reducing the avidity of HA for its receptor so that it can dissociate without the sialidase activity [187]. In this vein, Blick et al. reported that a zanamivir resistant virus incorporating an HA mutation that significantly impaired affinity, actually required the presence of the drug for viral growth, because returning functionality of the NA shifts the balance to overwhelming sialidase activity and HA cannot effectively complete the receptor binding and entry process [188]. Similarly, when NA is inherently defective, growth can be rescued by adaption of HA [189,190,191,192,193]. Baigent et al. combined the study of HA glycosylation and NA stalk length, both characteristics found to change among transmission and adaptation, and illustrated a fine balance of the two modifications needed to sustain infection [67]. As suggested by many, the functional balance of HA and NA has implications for not only the general fitness of the virus, but also interspecies transmission and virulence [152,194]. Though the need for functional balance has been well established, the timing of these interactions within the course of infection is still unknown. Studies specifically addressing the role of NA at the point of entry have revealed that NA activity could have an impact on the initiation of infection [195]. Novel techniques such as bio-layer interferometry have been developed and allow for measurement in real time of concurrent binding and enzymatic reactions to a sialic acid containing substrate [196]. Characterizing the interplay between the two proteins during all stages of infection, from prior to entry to release after budding, will facilitate a better understanding of the broad processes of viral host interaction.

## Figures and Tables

**Table 1:** Lectin histochemical staining of avian, swine and human cells.

Species	Tissue	2,3Sia	2,6Sia	Reference
Ducks	Trachea	+	+	Franca et al. [111], Costa et al. [114]
		+		Kuchipudi et al. [112]
	Bronchus/lung	+	+	Franca et al. [111], Kuchipudi et al. [112], Costa et al. [114]
	Intestine	+	+	Franca et al. [111]
		+		Kuchipudi et al. [112], Gambaryan et al. [116], Costa et al. [114]
Gulls	Trachea	+	+	Franca et al. [111]
	Bronchus/lung	+	+	Franca et al. [111]
	Intestine	+	+	Franca et al. [111]
Quail	Trachea	+	+	Wan et al. [113]
	Intestine	+	+	Wan et al. [113]
Chickens	Trachea	+		Wan et al. [113]
			+	Kuchipudi et al. [112], Trebbien et al. [117]
		+	+	Gambaryan et al. [116], Costa et al. [114]
	Bronchus/lung	+	+	Kuchipudi et al. [112], Gambaryan et al. [116], Costa et al. [114]
	Intestine	+		Kuchipudi et al. [112], Costa et al. [114], Wan et al. [113]
		+	+	Gambaryan et al. [116], Trebbien et al. [117]
Pigs	Trachea		+	Nelli et al. [118], Chan et al. [119], VanPoucke et al. [120]
	Bronchus/lung	+	+	VanPoucke et al. [120], Chan et al. [119], Punyadarsaniya et al. [121], VanPoucke et al. [120]
			+	Nelli et al. [118], Trebbien et al. [117]
Humans	Trachea	inmucindroplets	+	Couceiro et al. [122]
	Bronchus/lung	+	+	Barkhordari et al. [123]
		+lower	+upper	Shinya et al. [42]
		variable	+	Nicholls et al. [124]
